# Dynamics of insecticide resistance in malaria vectors in Benin: first evidence of the presence of *L1014S *kdr mutation in *Anopheles gambiae *from West Africa

**DOI:** 10.1186/1475-2875-10-261

**Published:** 2011-09-12

**Authors:** Innocent Djègbè, Olayidé Boussari, Aboubakar Sidick, Thibaud Martin, Hilary Ranson, Fabrice Chandre, Martin Akogbéto, Vincent Corbel

**Affiliations:** 1 Centre de Recherche Entomologique de Cotonou (CREC), 06 BP 2604, Cotonou, Bénin; 2 Institut de recherche pour le développement (IRD), Maladies Infectieuses et Vecteurs, Ecologie, Génétique, Evolution et Contrôle (MIVEGEC), UM1-CNRS 5290-IRD 224, 01 BP 4414 RP Cotonou, Bénin; 3 Centre de Coopération Internationale en Recherche Agronomique pour le Développement (CIRAD), UR-Hortsys, 34980 Montpellier, France; 4 Vector Group, Liverpool School of Tropical Medicine, Liverpool, L3 5QA, UK; 5 IRD, MIVEGEC, UM1-CNRS 5290-IRD 224, Laboratoire de lutte contre les Insectes Nuisibles (LIN), Montpellier France

## Abstract

**Background:**

Insecticide resistance monitoring is essential to help national programmers to implement more effective and sustainable malaria control strategies in endemic countries. This study reported the spatial and seasonal variations of insecticide resistance in malaria vectors in Benin, West Africa.

**Methods:**

*Anopheles gambiae s.l *populations were collected from October 2008 to June 2010 in four sites selected on the basis of different use of insecticides and environment. WHO susceptibility tests were carried out to detect resistance to DDT, fenitrothion, bendiocarb, permethrin and deltamethrin. The synergist piperonyl butoxide was used to assess the role of non-target site mechanisms in pyrethroid resistance. *Anopheles gambiae *mosquitoes were identified to species and to molecular M and S forms using PCR techniques. Molecular and biochemical assays were carried out to determine *kdr *and *Ace.1^R ^*allelic frequencies and activity of the detoxification enzymes.

**Results:**

Throughout the surveys very high levels of mortality to bendiocarb and fenitrothion were observed in *An. gambiae s.l*. populations. However, high frequencies of resistance to DDT and pyrethroids were seen in both M and S form of *An. gambiae s.s*. and *Anopheles arabiensis*. PBO increased the toxicity of permethrin and restored almost full susceptibility to deltamethrin. *Anopheles gambiae s.l*. mosquitoes from Cotonou and Malanville showed higher oxidase activity compared to the Kisumu susceptible strain in 2009, whereas the esterase activity was higher in the mosquitoes from Bohicon in both 2008 and 2009. A high frequency of *1014F kdr *allele was initially showed in *An. gambiae *from Cotonou and Tori-Bossito whereas it increased in mosquitoes from Bohicon and Malanville during the second year. For the first time the *L1014S kdr *mutation was found in *An. arabiensis *in Benin. The *ace.1^R ^*mutation was almost absent *in An. gambiae s.l*.

**Conclusion:**

Pyrethroid and DDT resistance is widespread in malaria vector in Benin and both metabolic and target site resistance are implicated. Resistance was not correlated with a change of malaria species and/or molecular forms. The *1014S kdr *allele was first identified in wild population of *An. arabiensis *hence confirming the expansion of pyrethroid resistance alleles in Africa.

## Background

Despite intense national and international efforts, malaria remains one of the major tropical challenges in the world today [1]. In Benin, the primary tools for malaria vector control are long-lasting insecticidal nets (LLIN) and indoor residual spraying (IRS). However, insecticide resistance development in vector populations could impede the success of malaria control programmes in endemic areas. In West Africa, the resistance of *Anopheles gambiae s.l*. to the four major classes of insecticides available for public health has been reported [2-4].

Pyrethroids are the only option for net treatment due to their relative safety for humans at low dosage, excito-repellent properties, rapid rate of knock-down and killing effects [5].

Resistance to this insecticide class is now widespread in the main malaria vectors *An. gambiae s.l, Anopheles arabiensis *and *Anopheles funestus *[6-9]. Both enhanced detoxification [10,11] and mutations in the gene encoding the voltage-gated sodium channel [12] have been shown to be important resistance mechanisms. The Leucine to Phenylalanine substitution at position 1014 (*L1014F*) was found predominant in West and central Africa [7,13] whereas the Leucine to Serine substitution (*L1014S*), originated from Kenya [14], has now spread in the central region including Cameroon [15,16], Equatorial Guinea [17], Gabon [18], Angola [17], Uganda [19] and Ethiopia [8].

The impact of this resistance on pyrethroid-based control is largely unquantified. Until recently, pyrethroid resistance based on *kdr *mutation or metabolic mechanisms in *An. gambiae *in Côte d'Ivoire [20,21] and Kenya [22,23], did not adversely affect the efficacy of pyrethroid-treated nets. However, a longitudinal survey recently conducted in southern Benin showed neither reduction of asymptomatic infection nor malaria attack by the use of LLINs in an area of pyrethroid resistance [24].

In Benin recent (2006-2007) entomological surveys reported cross-resistance to DDT and pyrethroids in *An. gambiae s.l*. with strong geographic variations in a south-north transect [13,25]. Molecular studies showed the presence of the *kdr L1014F *mutation and an overexpression of two P450 genes (CYP6M2 & CYP6P3) potentially involved in pyrethroid resistance [26]. The presence of a single point mutation (glycine to serine at position 119) in the oxyanion hole of the acetylcholinesterase enzyme [3,27,28] conferring resistance to carbamates and to a lesser extend to organophosphates was also detected in Burkina-Faso, Côte d'Ivoire and Benin [13,29,30].

The intense use of DDT in agricultural settings and during the WHO malaria eradication programme in the 1950s and 1960s were suspected to be the main factors selecting for pyrethroids and DDT resistance in *An. gambiae *populations [31]. Various insecticidal products (organophosphates, pyrethroids, etc.) are also used for crop protection but the amount applied is far higher than that consumed in public health against malaria vectors [2]. Benin is still an important producer of cotton in West Africa and 90% of pesticide products are directed against cotton pests [32,33]. Small-scale vegetable farming is an important source of livelihood in urban and peri-urban environments [34] and provides income and food for tens of thousands of families [35]. Akogbéto *et al *[31,36] reported that mosquito species, *An. gambiae *in particular, lay their eggs in breeding sites located around agricultural settings. These eggs undergo a selection pressure from agricultural pesticides, which leads to the emergence of resistant populations of *An. gambiae*, thereby impeding malaria vector control operations.

The Beninese National Malaria Control Programme received financial and technical support from World Bank, Global fund and WHO to implement large-scale and free distribution of LLIN since 2007 [37]. Several authors have studied the effect of insecticide treated nets (ITNs) with pyrethroids on *An. gambiae *populations and the possible selection of *kdr *alleles either in laboratory experiments [38] or experimental huts trials [39] or in the field [40,41]. Increasing resistance of malaria vectors may have important implications for vector control programmes, especially considering the scaling up of LLINs and IRS in Africa. Hence, knowledge on spatio-temporal changes in insecticide resistance level is a basic requirement to guide the use of insecticides in malaria control programmes.

In this study, the dynamic of insecticide resistance was evaluated in *An. gambiae *populations collected in four sentinel sites selected on the basis of different agricultural practices, use of insecticides and environment (urban/rural areas). From 2008 to 2010, temporal changes in insecticide resistance level, sibling species among *An. gambiae s.l*, enzymatic activity and frequency of resistant alleles were measured twice per year through a combination of insecticide bioassay, biochemical and molecular techniques.

## Methods

### Study area

**Figure 1:**
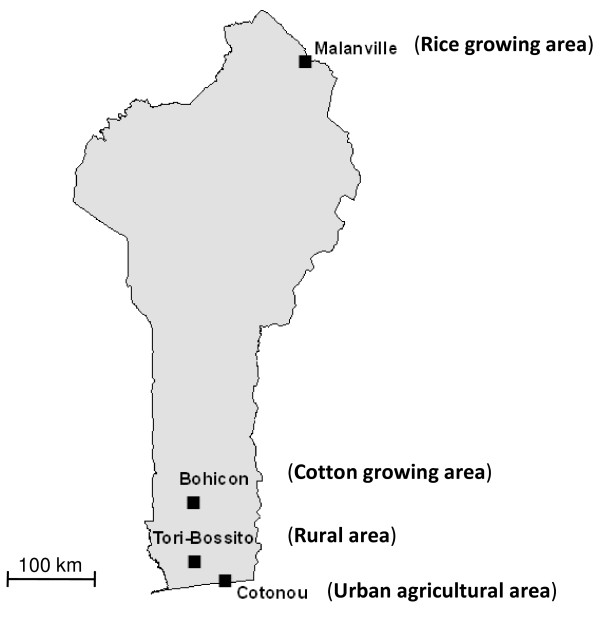
**Map of Benin showing the mosquito collection sites**.

Four representative sites in Benin (Figure 1) were selected for the biannual resistance monitoring on the basis of expected insecticide selection pressures. These sites are Asecna, Tori-Bossito, Bohicon and Malanville. Asecna (6°21N-2°23E) is a 14 ha vegetable growing area located in Cotonou and where locals apply uncontrolled amount of insecticides for crop protection (cabbages, lettuces and tomatoes) [31]. Tori-Bossito (2°89E-6°30N) is located in the South-West of the country where farmers cultivate maize and cassava and where people used ITN and sprays for protection against malaria vectors. Bohicon (2°49E - 7°11N) is located in the middle part of the country, where the farmers used significant amounts of pyrethroids and organophosphates for cotton protection. Malanville (11°52N- 3°23E) is a 100 ha rice growing area located in the far north of Benin, near the Niger River, where the insecticide use is minimal for rice protection.

### Mosquito collection

From October 2008 to June 2010, larvae of *An. gambiae *mosquito were collected twice per year, i.e. at the beginning and the end of rainy season from a wide range of breeding sites (puddles, shallow wells, gutters and rice fields). In each locality, larval collections were carried out in, at least, 15 breeding sites in which an average of 100 larvae (all instars) per habitat were collected. All larvae were brought back to laboratory of Centre de Recherche Entomologique de Cotonou (CREC) for rearing. Emerging adult female mosquitoes (F_0_) were used for insecticide susceptibility tests. A susceptible strain of *An. gambiae *(Kisumu) was used as reference strain for bioassays and biochemical studies.

### Insecticide susceptibility test

WHO insecticide susceptibility test-kits and standard procedures [42] were used to monitor the susceptibility of wild *An. gambiae *populations to the four chemical groups of insecticides commonly used in public health and agriculture. *Anopheles gambiae s.l*. mosquitoes were first morphologically identified and then submitted to bioassays. Batches of 25 non-blood fed, 3-5 days old adult females were exposed to filter papers impregnated with 4% DDT (organochlorine), 1% fenitrothion (organophosphate), 0.1% bendiocarb (carbamate), 0.75% permethrin and 0.05% deltamethrin (pyrethroids). Insecticide papers were obtained from the WHO reference centre at the Vector Control Research Unit, University Sains Malaysia [43]. For each test, 100 mosquitoes were exposed to treated and untreated (control) papers for 1 hour. The piperonyl butoxide (PBO), an inhibitor of oxidases and esterases, was used as a synergist of pyrethroids whenever possible. In this case, mosquitoes were first exposed to PBO (4%) during 1 hour and then to permethrin or deltamethrin as described above. The mortality was recorded after 24 hour. WHO criteria were followed to classify populations as 'resistant' if less than 80% mortality was observed, as ''suspected resistant'' if mortality rates were between 80 and 97% and susceptible for mortality > 97% [43].

### Biochemical analysis

Biochemical assay were used to quantify levels of oxidase, non-specific esterase and GST activities in individual 2-5 days old adults of *An. gambiae *that have not been previously exposed to insecticides (i.e. control batches). A total of 47 specimens were tested per microtitre plate according to the method described by Hemingway [44]. Each plate contained 10 unfed adult mosquitoes from the susceptible Kisumu strain to allow for comparison with field mosquito populations.

### Molecular identification

For each sentinel site, 30-50 mosquitoes taken from the control tubes were identified to species using PCR [45] and as M and S molecular forms by PCR-RFLP technique [46].

### PCR detection of the Kdr Leu-Phe, Leu-Ser and Ace.1 mutations

The presence of *1014F *and *1014S kdr *alleles was tested on mosquitoes from bioassay control using HOLA (Hot Oligonucleotide Ligation Assay) technique according to protocol of Lynd et al [47]. All samples positive for the 1014S allele were sent for sequencing to confirm the results. The PCR-FRLP diagnostic test was used to detect the presence of G119S mutation (*Ace.1 *gene) as described by Weill *et al *[48].

### Data analysis

Data of mortality rates obtained with or without synergist were compared using a Chi-square test with the MINITAB statistical software. Biochemical assay data (enzymatic activity per mg protein) were compared between the reference strain (Kisumu) and the field-caught populations by a Kruskal-Wallis non-parametric test. The frequency of resistant alleles (*kdr *and *ace.1^R^*) was compared by chi-square test implemented using GENEPOP software between species (*An. gambiae *M and S forms and *An. arabiensis*) and round collections [49].

## Results

### Resistance status

**Figure 2:**
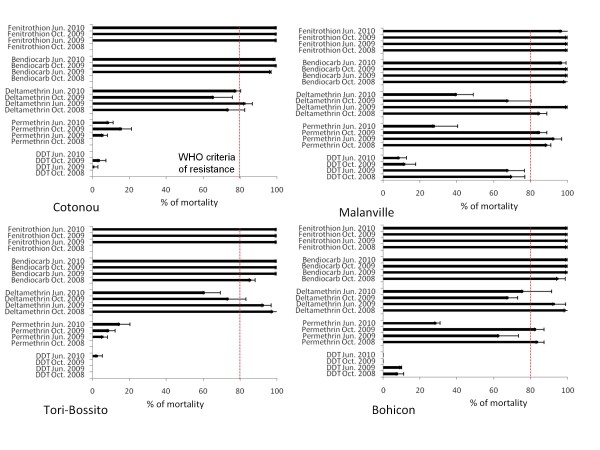
**Insecticide susceptibility status of *Anopheles gambiae s.l*. in the four sentinel sites in Benin**. Error bars are standard deviations. Nota: In October 2008 because of lower number of mosquitoes collected susceptibility tests were not performed with all five insecticides in Cotonou (only deltamethrin was tested) and Tori-Bossito (only deltamethrin and bendiocarb were tested).

Figure 2 shows the insecticide resistance status of four *An. gambiae s.l *populations collected in Benin during 4 collection rounds (2008 to 2010). Throughout the surveys, the susceptible strain Kisumu of *An. gambiae *displayed mortality rates above 98% for the five insecticides tested. In control groups (untreated papers) mortality rates of wild *An. gambiae *populations were always below 10% 24 hours post-exposure.

*Anopheles gambiae s.l *populations collected in the four sites remained almost fully susceptible to bendiocarb and fenitrothion (Figure 2). Conversely, high frequencies of DDT and permethrin resistance were initially found at Cotonou and Tori-Bossito. A significant decrease in permethrin mortality rates was recorded between 2009 and 2010 in Malanville and Bohicon (p < 0.0001) but DDT resistance was already high in the latter site. Bioassays showed a global increase of deltamethrin resistance throughout the two years surveys in Tori-Bossito, Bohicon and Malanville (p < 0.0001).

### Synergist and biochemical analysis

**Figure 3:**
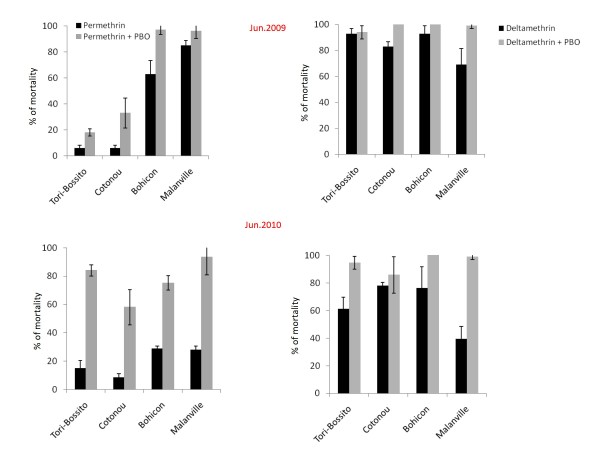
**WHO cylinder tests showing the efficacy of pyrethroids in two collection rounds in the four sentinel sites with and without pre-exposure to the synergist PBO (Error bars are standard deviations)**.

Figure 3 shows the insecticidal activities of permethrin and deltamethrin against *An. gambiae s.l*. mosquitoes (June 2009 and June 2010) with and without the synergist PBO. Pre-exposure of mosquitoes to PBO significantly increased the mortality rates of permethrin and to a lesser extend deltamethrin at Cotonou, Bohicon and Malanville (p < 0.05) in 2009 whereas in 2010, PBO strongly enhanced the mortality rates of deltamethrin especially in Tori-Bossito and Malanville (p < 0.0001).

**Figure 4:**
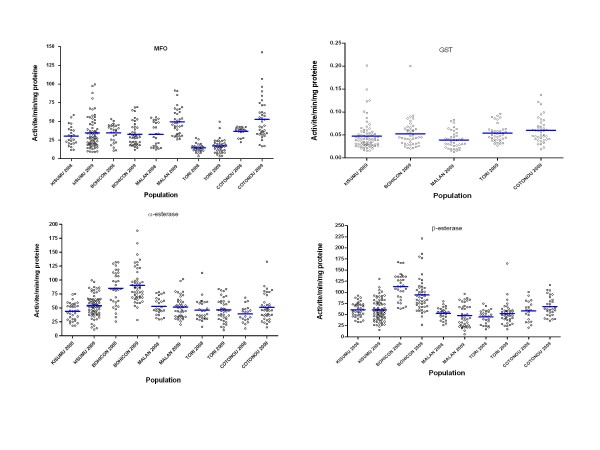
**Variation of MFO, NSE (with substrate alpha and beta-naphthyl acetate) and GST activities per individuals of *Anopheles gambiae s.l*. collected in the four sentinel sites from 2008 to 2009, compared with the susceptible reference strain kisumu**.

Data from biochemical analyses carried out in 2008 and 2009 (once per year: October) are shown in Figure 4. *Anopheles gambiae s.l*. mosquitoes collected in Cotonou and Malanville in 2009 showed significantly higher oxidase activity (P < 0.05) compared to those of the susceptible reference strain Kisumu. Higher activity of esterase (using both α-naphtyl and β-naphtyl acetate as substrates) was observed in Bohicon samples in both years compared to Kisumu and other field populations (P < 0.0001) (Figure 4).

### Species and molecular forms of *Anopheles gambiae*

**Figure 5:**
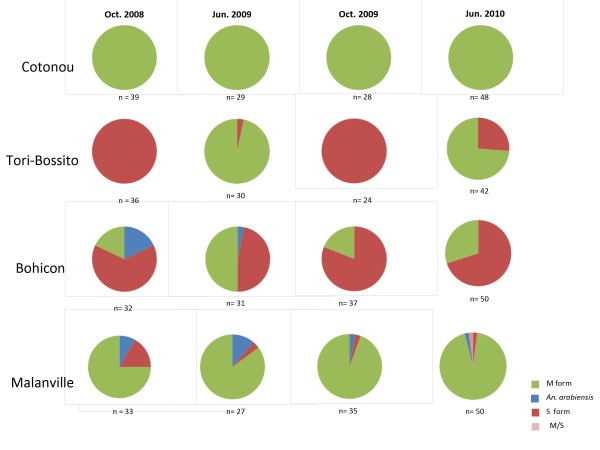
**Species composition in each sentinel site per round collection**. *An. gambiae s*amples from control bioassays were identified to species and molecular form by PCR.

Mosquitoes from bioassay (i.e. control batches) were analysed by PCR for identification of sibling species among *An. gambiae s.l*. complex and molecular M and S forms of *An. gambiae s.s*. In all sites, *An. gambiae s.s *was predominant over *An. arabiensis *(Figure 5). Only *An. gambiae s.s*. was found in Cotonou and Tori-Bossito while some *An. arabiensis *were caught in Bohicon (18% in October 2008 versus 3% in June 2009) and Malanville (8% in October 2008 versus 11% in June 2009). The molecular M form of *An. gambiae *was predominant in Cotonou (urban costal area) and Malanville (rice field) whereas both M and S forms were found in sympatry in Tori-Bossito and Bohicon. A switch from M to S form occurred at the end of the rainy season (October) especially in Tori-Bossito, hence suggesting important difference in the preference of ecological niches between the two species.

### Detection of resistance genes

The *L1014F kdr *mutation was found in *An. gambiae s.s *(both M and S) and *An. arabiensis *but at various allelic frequencies (see Table 1). The *1014F *allelic frequency was high and almost fixed in the M form in Cotonou. In Bohicon and Malanville, the *1014F *allelic frequency increased significantly in M form from October 2008 to June 2010 (p < 0.0001). This increase of *kdr *allelic frequency was positively correlated with bioassays data. The same was true at Tori-Bossito where the *1014F kdr *allele increased from October 2008 to October 2010 (p < 0.0001). Because of low number of *An. arabiensis *collected, the *1014F *allelic frequencies were not compared between the sites and round collections.

**Table 1:** Kdr *1014F and 1014S *frequencies in *An.gambiae s.l *per round collection and locality

*Anopheles gambiae s.l*	Sites	Periods	N	Leu-Leu	Leu-Phe	Leu-Ser	Phe-Phe	Ser-Ser	F(1014F)	F(1014S)
M Form	Cotonou	October 2008	39	3	5	-	31	-	0.85^a^	-
		June 2009	29	-	5	-	24	-	0.91^a^	-
		October 2009	28	-	1	-	27	-	0.98^b^	-
		June 2010	46	-	9	-	37	-	0.90^a^	-
	Tori-Bossito	October 2008	-	-	-	-	-	-	-	-
		June 2009	30	-	5	-	25	-	0.91^c^	-
		October 2009	-	-	-	-	-	-	-	-
		June 2010	31	-	14	-	16	-	0.77^d^	-
	Bohicon	October 2008	7	5	1	-	1	-	0.21^e^	-
		June 2009	16	-	7	-	9	-	0.78^f^	-
		October 2009	7	-	1	-	6	-	0.92f^g^	-
		June 2010	15	-	1	-	11	-	0.76^f^	-
	Malanville	October 2008	27	20	5	-	2	-	0.16^h^	-
		June 2009	26	18	7	-	1	-	0.17^h^	-
		October 2009	34	20	8	-	6	-	0.29^i^	-
		June 2010	44	12	20	-	12	-	0.50^j^	-

S Form	Tori-Bossito	October 2008	36	8	12	-	16	-	0.61^k^	-
		June 2009	1	-	1	-	-	-	0.50^k^	-
		October 2009	24	-	4	-	20	-	0.91^l^	-
		June 2010	11	1	5	-	5	-	0.68^k^	-
	Bohicon	October 2008	25	3	6	-	16	-	0.76^m^	-
		June 2009	15	-	1	-	14	-	0.96^n^	-
		October 2009	30	-	3	-	27	-	0.95^n^	-
		June 2010	33	-	7	-	26	-	0.89^mn^	-
	Malanville	October 2008	7	5	1	-	1	-	0.21	-
		June 2009	1	1	-	-	-	-	-	-
		October 2009	1	1	-	-	-	-	-	-
		June 2010	1	-	-	-	1	-	1.00	-

*An. arabiensis*	Bohicon	October 2008	7	1	4	-	2	-	0.57	-
		June 2009	1	-	-	-	1	-	1.0	-
		October 2009	0	-	-	-	-	-	-	-
		June 2010	2	1	-	-	-	1	0.5	0.50
	Malanville	October 2008	3	3	-	-	-	-	-	-
		June 2009	3	3	-	-	-	-	-	-
		October 2009	1	1	-	-	-	-	-	-
		June 2010	3	-	1	2	-	-	0.16	0.33

'-' indicates no samples available for this round collection; Leu = Leucine, Phe = Phenylalanine, Ser = Serine. Nota: A further two individuals with the 1014S allele including one homozygote and one heterozygote were respectively detected in Bohicon and Malanville but their species could not be confirmed. The numbers carrying the same letter are not significantly different

The *1014S kdr *allele was detected for the first time in Benin (Malanville and Bohicon in June 2010). The *1014S *kdr allele was identified in five *Anopheles gambiae s.l *mosquitoes. Among them, three mosquitoes were identified as *An. arabiensis *including two heterozygote from Malanville and one homozygote from Bohicon. The two other 1014S positive specimens could not be clearly identified for species. The *Ace.1^R ^*allele was only found in the heterozygote state in one specimen of *An. gambiae *M form in Bohicon.

## Discussion

Information on the resistance status of the main malaria vectors is essential to guide the choice of insecticides to use by National Malaria Control Programmes. In this context, a WHO/TDR programme has been launched in five countries (Chad, Angola, Sudan, Burkina Faso and Benin) to assess the spatio-temporal variation of insecticide resistance in the main malaria vectors [6]. One of the major objectives of this network was to monitor insecticide resistance in malaria vectors in prioritized areas where large-scale insecticide based control programmes are being implemented or planned by national authorities.

Regarding the distribution of malaria vector species, *An. gambiae s.s *was predominant in all sites, followed by *An. arabiensis*. This is consistent with a previous studies carried out in 30 localities in Benin [50]. Consistent temporal variation in sibling species of *An. gambiae *was observed in 2 of the 4 sentinel sites. The M form was predominant in Cotonou and Malanville whereas both molecular M and S forms were found in sympatry in Bohicon and Tori-Bossito with important seasonal variation in their relative abundance. Interestingly, the M form was entirely replaced by the S form at Tori-Bossito, at the end of the rainy season. This may be explained by the fact that the relative dominance of one molecular form over the other is strongly associated with a specific and characteristic breeding site [51]. Indeed, the M form in Tori-Bossito is mainly associated with domestic permanent breeding sites (i.e. water storage containers such as water tank, jars and barrels) whereas the S form is associated with rain-dependent temporary sites [52,53]. However, it seems that the replacement of the M form by the S didn't impact on the level of DDT and pyrethroid resistance as well as on the prevalence of the *kdr *allele i.e. the *kdr *mutation was comparable between the two molecular forms (P > 0.05).

In Benin, the level of cross resistance between permethrin and DDT in *An. gambiae s.l*. was strong and correlated with the presence of *kdr *mutations and enhanced metabolic detoxification as shown by the synergist studies. Significant change in insecticide resistance and prevalence of the *kdr *alleles were observed throughout the study. Throughout the 2 years surveys, the level of DDT and pyrethroid resistance (permethrin and deltamethrin) strongly increased in field populations, hence confirming the global augmentation of pyrethroid resistance in African malaria vectors [54]. However, important differences were noted according to the ecological setting and round collection. In Cotonou, where large amount of insecticides are applied in urban vegetable farming [31], the *kdr *frequency was high and almost fixed whatever the round collection. Enhanced detoxification process due to higher oxidase activity was detected and seemed to play important role in resistance to DDT, permethrin and to a lesser extend deltamethrin [26]. In Bohicon, a cotton growing area where various insecticidal products (organophosphates, pyrethroids, etc.) are used to control agricultural pests, prevalence of the *kdr *mutation increased between 2008 and 2010 precisely in the M form. Higher activity of non-specific esterase was also detected but their role in insecticide detoxification need to be clarified. This result may reflect an increase selection pressure on malaria vectors due to agricultural practices [55,56]. Based on strong supporting results, several authors [56-59] hypothesized that past and current agricultural use of pyrethroids, DDT and organophosphates for crop protection led to the selection of resistant individuals by challenging larval stages with residual insecticide products accumulating in water bodies around cultivated areas. Indeed, Akogbéto *et al *[36] demonstrated in Benin that pyrethroid resistant *An. gambiae *larvae reared in water and soil samples taken from vegetable gardens or cotton area were able to survive and proliferate in contrast to the susceptible phenotype. In Tori-Bossito, the level of DDT and permethrin resistance as well as the prevalence of the *kdr *frequency was initially high. However, intense malaria vector control programmes were implemented since 2007 (i.e. increased coverage of LLIN at community level), and the *kdr *frequency increased from 0.34 in 2007 [28] to more than 0.77 in 2010 among both M and S molecular forms. Knowing the different larval habitats preferences between the M and S form in this locality [60], it's likely that vector control intervention based on LLIN distribution has led to increase the level of pyrethroid resistance and select for *kdr *alleles in malaria vectors. This is consistent with previous observations reporting an increase of the *kdr 1014F *frequency in *An. gambiae *following a nationwide distribution of long-lasting insecticide-treated nets in Niger and Kenya [41,61]. In the rice field area of Malanville, the increase in resistance to DDT and pyrethroids can reasonably be attributed to the augmentation of the *1014F kdr *prevalence and to higher levels of oxidase activity. Corbel *et al *[27] previously reported a very low frequency of the *kdr 1014F *frequency (0.06 in 2007) whereas in 2010, this frequency has reached 50% in the M form. The sudden increase in *kdr *frequency and pyrethroid resistance is worrying considering the relatively low amount of insecticide use in this area. It is possible that *An. gambiae *populations carrying the *kdr *mutation might have migrated, through active or/and passive ways, from bordering countries (e.g. Niger, Nigeria) due to intense traffic and exchanges in this locality. Experimental huts belonging to the ABC (Anopheles, Biology and Control) network were initially built in Malanville to assist WHOPES (WHO Pesticide Evaluation Scheme) in testing and evaluation of insecticide products against susceptible malaria vectors. The increasing resistance pattern in Malanville highlights the difficulty in finding areas where the vector population remain pyrethroid-susceptible to conduct such trials. It is obvious that WHOPES will have to consider this trend in the development of future guidelines for testing insecticide products for IRS and LLIN.

An important result of the present study is the first report of the *L1014S kdr *mutation in wild *An. gambiae s.l *populations from Benin. The resistant allele was found in *An. arabiensis*. This allele has been reported in *An arabiensis *in Uganda [19] and Ethiopia [8]. The 1014S allele is also found in *An gambiae s.s*. of the M and S forms in Central Africa (Equatorial Guinea [62] and Cameroon [63]) but its presence in this species was not confirmed in the current study. Among the five specimens of mosquitoes sharing the *1014S *allele, three were found at the homozygote state and two at the heterozygote. These findings provide additional evidence of the rapid spread of *kdr *mutations in *An. gambiae s.l *throughout Africa and will serve as baseline data for careful monitoring this allele in West African countries. Further studies are need to determine the geographical distribution of *1014S kdr *allele in West Africa, its role in pyrethroid phenotypic resistance and its impact on the efficacy of pyrethroid treated materials.

Encouragingly, this study demonstrated the full susceptibility of malaria vectors to fenitrothion and bendiocarb, hence confirming the work of Djogbenou *et al *[13] in Benin. This susceptibility was associated with the virtual absence of the *Ace.1^R ^*allele in the four sentinel sites. This may be explained by low selection pressure on this resistant allele in Benin and/or strong genetic cost associate with altered acetylcholinesterase [64]. This result is important for vector control since carbamates and organophosphates are regarded as potential alternatives to pyrethroids for IRS as part of an insecticide resistance management strategy [65,66]. Indeed, these insecticides could be used alone or in rotation with others new insecticides, such as chlorfenapyr, to counteract the development of pyrethroid resistance [67].

## Conclusion

These results showed a seasonal and spatial evolution of multiple insecticide resistance in malaria vectors in Benin that may seriously compromise malaria vector control efforts implemented in the country since 2007. This study showed first evidence for the presence of the *1014S kdr *allele in wild *An. arabiensis *populations from Benin and West Africa and highlights the urgent need to continue monitoring of insecticide resistance in African malaria vector. The development of new tools and strategies for pyrethroid-resistance management are urgently needed.

## Competing interests

The authors declare that they have no competing interests.

## Authors' contributions

ID carried out the larval collection, bioassay. ID and AS carried out the biochemical and molecular analysis. ID and VC drafted the manuscript. VC, TM, FC, HR and MA participated to the design of the study. ID and OB participated to the data analysis. All authors read and approved the final manuscript.
